# Clinical Features Predicting Mortality Risk in Patients With Viral Pneumonia: The MuLBSTA Score

**DOI:** 10.3389/fmicb.2019.02752

**Published:** 2019-12-03

**Authors:** Lingxi Guo, Dong Wei, Xinxin Zhang, Yurong Wu, Qingyun Li, Min Zhou, Jieming Qu

**Affiliations:** ^1^Department of Respiratory and Critical Care Medicine, Ruijin Hospital, Shanghai Jiao Tong University School of Medicine, Shanghai, China; ^2^Institute of Respiratory Diseases, Shanghai Jiao Tong University School of Medicine, Shanghai, China; ^3^Research Laboratory of Clinical Virology, Ruijin Hospital, Shanghai Jiao Tong University School of Medicine, Shanghai, China; ^4^Department of Infectious Diseases, Institute of Infectious and Respiratory Diseases, Ruijin Hospital, Shanghai Jiao Tong University School of Medicine, Shanghai, China; ^5^Clinical Research Center, Ruijin Hospital North, Shanghai Jiao Tong University School of Medicine, Shanghai, China; ^6^Department of Respiratory Medicine, The Third People’s Hospital of Zhengzhou, Henan, China

**Keywords:** virus pneumonia, predicting mortality, bacterial coinfection, predictive score model, clinical feature

## Abstract

**Objective:**

The aim of this study was to further clarify clinical characteristics and predict mortality risk among patients with viral pneumonia.

**Methods:**

A total of 528 patients with viral pneumonia at RuiJin hospital in Shanghai from May 2015 to May 2019 were recruited. Multiplex real-time RT-PCR was used to detect respiratory viruses. Demographic information, comorbidities, routine laboratory examinations, immunological indexes, etiological detections, radiological images and treatment were collected on admission.

**Results:**

76 (14.4%) patients died within 90 days in hospital. A predictive MuLBSTA score was calculated on the basis of a multivariate logistic regression model in order to predict mortality with a weighted score that included multilobular infiltrates (OR = 5.20, 95% CI 1.41–12.52, *p* = 0.010; 5 points), lymphocyte ≤ 0.8^∗^10^9^/L (OR = 4.53, 95% CI 2.55–8.05, *p* < 0.001; 4 points), bacterial coinfection (OR = 3.71, 95% CI 2.11–6.51, *p* < 0.001; 4 points), acute-smoker (OR = 3.19, 95% CI 1.34–6.26, *p* = 0.001; 3 points), quit-smoker (OR = 2.18, 95% CI 0.99–4.82, *p* = 0.054; 2 points), hypertension (OR = 2.39, 95% CI 1.55–4.26, *p* = 0.003; 2 points) and age ≥60 years (OR = 2.14, 95% CI 1.04–4.39, *p* = 0.038; 2 points). 12 points was used as a cut-off value for mortality risk stratification. This model showed sensitivity of 0.776, specificity of 0.778 and a better predictive ability than CURB-65 (AUROC = 0.773 vs. 0.717, *p* < 0.001).

**Conclusion:**

Here, we designed an easy-to-use clinically predictive tool for assessing 90-day mortality risk of viral pneumonia. It can accurately stratify hospitalized patients with viral pneumonia into relevant risk categories and could provide guidance to make further clinical decisions.

## Statement

Viral infections could present with severe pneumonia, acute respiratory distress syndrome or are complicated by bacterial super-infections in many patients. Influenza and other respiratory viruses are common reasons of acute pneumonia which can result in significant morbidity or mortality in the setting of high-risk factors such as extremes of age, pregnancy, obesity or chronic pre-existing conditions. Cytokines and chemokines, on the other hand, are regarded as possible hallmarks of severe disease and many of them reach high serum levels in the setting of severe infection. Although a variety of clinical prediction rules for pneumonia such as CRB-65 and CURB-65 are widely used in the assessment of community acquired pneumonia, no standard rule for the calculation of viral pneumonia severity scores has been established to our knowledge. Here, we designed a easy-to-use clinically predictive score for assessing mortality risk of viral pneumonia. This model showed better predictive ability with a c-index of 0.811, sensitivity of 0.776 and specificity of 0.778. A cut-off value of 12 points could be used for mortality risk stratification.

## Background

Viral infections, in spite of their common manifestations as mild illnesses, present with severe pneumonia, acute respiratory distress syndrome (ARDS) or bacterial coinfections in many patients (Shorr et al., 2017). In recent years, the dissemination of PCR has increased the ability to detect respiratory viruses in both upper and lower-respiratory tract samples (Das et al., 2015). Influenza and other respiratory viruses are common reasons of acute respiratory infection. Patients predisposed to bacterial infections have greater morbidity and mortality levels (Hanada et al., 2018).

During natural infection, both the adaptive and innate immune responses play important roles in controlling respiratory virus infection (Nussing et al., 2018). Adaptive T and B cells maintain immunological memory and provide protection against subsequent virus infections. Cytokines and chemokines, on the other hand, are regarded as possible hallmarks of severe disease and many of them reach high serum levels in the setting of severe infections (La Gruta et al., 2007). Such variables also could guide clinical decision making as well as infectious disease management.

Although a variety of clinical prediction rules for pneumonia such as CRB-65 and CURB-65 are widely used in the assessment of community acquired pneumonia (CAP) (Viasus et al., 2016; Uranga et al., 2018), most remain not applicable in the setting of viral infection. Other reported risk factors for influenza pneumonia such as PO2/FiO2, lymphocyte count, and antigen-specific T cells are likewise useful in predicting mortality and deciding on appropriate management (Viasus et al., 2011; Shi et al., 2017). To our knowledge, no standard rule for the calculation of viral pneumonia severity scores has been established.

Here, we aimed to further elucidate the potential risk factors and attempt to predict the probability of mortality among patients infected with respiratory viruses.

## Materials and Methods

### Study Design and Population

A retrospective single-center observational study was conducted from May 2015 to May 2019 in RuiJin Hospital, Shanghai, China. The study was approved by Ruijin Hospital Ethics Committee and written informed consent was obtained from all patients involved before enrolment.

We retrospectively studied all hospitalized patients with positive result of multiplex real-time reverse-transcription polymerase chain reaction (RT-PCR, TIB respiratory kit, ROCHE, Switzerland) aiming to detect respiratory viruses. The time period of this study was selected because of the introduction of the viral test panel. Patients who were diagnosed pneumonia according to the 2009 Infectious Diseases Society of American (IDSA)/American Thoracic Society (ATS) guidelines (Charles et al., 2009) were enrolled in this study. Patients were excluded if: 1) age <18 years; 2) had a clear alternative final diagnosis as lung cancer or other non-pneumonia illness; 3) long hospitalization >3 months before death.

**FIGURE 1:**
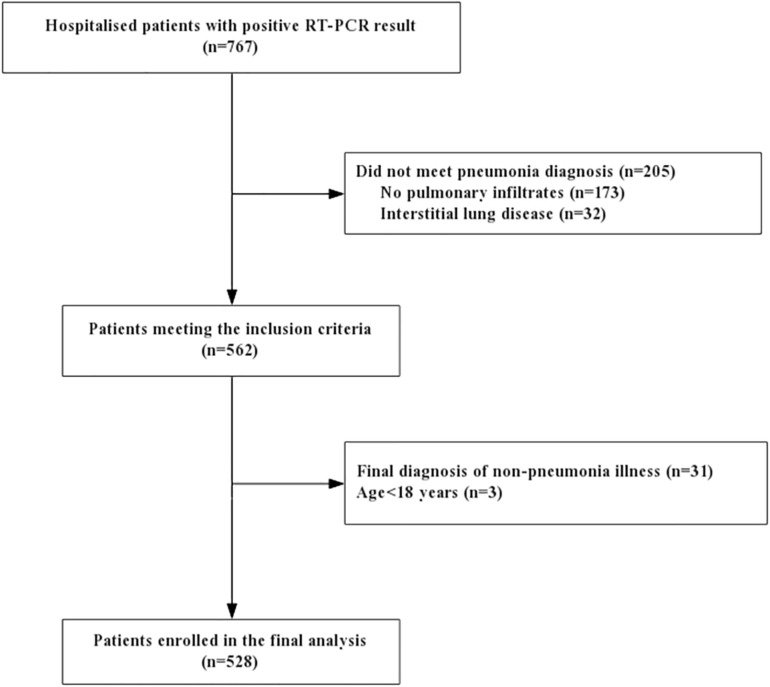
Study flowchart. RT-PCR: reverse-transcription polymerase chain reaction.

767 hospitalized patients had initial positive RT-PCR results and 562 of them were enrolled with pneumonia. A total of 34 cases were excluded: final diagnosis of non-pneumonia illness (*n* = 31), children or adolescent patient (*n* = 3). 528 pneumonia patients with positive viral detection were finally included in this analysis (Figure 1).

### Data Collection

Infections due to influenza A (FluA), adenovirus (AdV), bocavirus, human rhinovirus (HRV), influenza B (FluB), parainfluenza (PIV), coronavirus (CoV), respiratory syncytial virus A (RSVA), respiratory syncytial virus B (RSVB), enterovirus (EV) and human metapneumovirus (HMPV) were confirmed using RT-PCR via nasal wash products. Data were collected on admission including demographic information, comorbidities, routine laboratory examinations, chest radiography or CT scanning, immunological and etiological detections. We used positive bacterial culture of blood and sputum samples as the criteria for bacterial growth. The use of antiviral therapy and steroids was recorded, including the drug, start date, duration and dosage. Patients were evaluated as deemed clinically appropriate at any time when pneumonia was suspected. CURB-65 score of each patient was calculated (Barlow et al., 2007). Length of stay and outcome state of each patient were recorded. Those improved patients with hospital stay <90 days were followed up by a phone call to determine survival status if they were not seen in the outpatient clinic. Finally, the outcome of mortality was defined as overall mortality within 90 days.

### Statistical Methods

Viral pneumonia patients were classified into two groups: survival group and 90-day death group. Univariate analysis was initially used to compare risk factors for mortality separately among patients with viral pneumonia. Proportions or means with SD were used to characterize the patient sample. Continuous variables were compared using *t*-tests or one-way ANOVA while χ2 or Fisher exact tests were used for categorical dependent data analysis, as appropriate.

The percentages of missing values of variables in our cohort were lower than 50%. We imputed missing data of the covariates by using multiple imputations (Sterne et al., 2009). Conclusions of univariate logistic regression analyses with or without imputed data were unchanged. Continuous variables were categorized and retained for multivariate testing. Cut-off points were identified following Youden’s index of receiver operator characteristic (ROC) curve or a clinically relevant cut-off. Variables with *p* < 0.10 were regarded as potential risk factors and included in multivariate regression analysis against overall mortality reduced by a backward elimination procedure (conditional likelihood ratio test and elimination if *p* ≥ 0.05).

Data of 528 patients was partitioned randomly into two complementary subsets: the training set of 423 (80%) was used to establish the model; the testing set of 105 (20%) was used to validate the analysis. For pragmatic reasons, scores for each predictors were assigned as integer values relative to the regression coefficient. Cut-off points were identified following Youden’s index of ROC. Survival analysis was performed using univariate approach with Kaplan-Meier analysis between low-risk and high-risk group according to the cut-off value.

Performance of the score was assessed by measuring the area under ROC curve (AUROC) while sensitivity and specificity were calculated. Internal validation was assessed by AUROC of 2000 bootstrapped samples. The cross-validation was assessed by calculating AUROC of the testing set. ROC curve and net reclassification improvement (NRI) (Leening et al., 2014) analyses were used to assess the improvement in risk predicting capacity compared with CURB-65. Statistical analysis was performed using SPSS version 22.0 and R 3.5.0. All tests were two-sided and a *p*-value <0.05 was considered significant.

## Results

### Patients Characteristics

Baseline characteristics of complete cases and different groups are described in Table 1. The mean age of viral pneumonia patients was 63.56 (SD 19.08) years and 61.2% were male. Approximately a third of patients had smoking history (31.8%, among them 10.8% had quit smoking before infection). A total of 360 (68.4%) patients had comorbidities, with hypertension the most commonly observed (49.8%), followed by diabetes, renal disease, coronary heart disease and chronic obstructive pulmonary disease (COPD). Overall, 141 patients (26.7%) had bacterial coinfection, 25 (4.8%) had fungi infection and 11 (2.1%) had tuberculosis. As for treatment, 166 patients (31.6%) received oseltamivir the first 48 h on admission. 144 patients (27.3%) were admitted to intensive care unit (ICU). The mean length of hospital stay in viral pneumonia patients was 20.72 (SD 20.25) days and hospital cost was 55644.36 (SD 93022.15) yuan.

**TABLE 1:** Population description and comparison between survivors and those who died in 90-days after admission.

	**Total population, *n* = 528**	**Survivors, *n* = 452**	**Died, *n* = 76**	***p-*value**^ 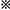 ^
**Socio-demographic details**				
Age (years)	63.56 ± 19.08	63.23 ± 19.58	65.47 ± 15.82	0.344
≥60^#^	371 (70.3)	308 (68.1)	63 (82.9)	**0.009**
Male^#^	323 (61.2)	270 (59.7)	53 (69.7)	0.092
Smoking^#^				**<0.001**
Acute-smoker	111 (21)	83 (18.4)	28 (36.8)	
Quit-smoker	57 (10.8)	47 (10.4)	10 (13.2)	
Non-smoker	360 (68.2)	322 (71.2)	38 (50)	
Occupation^#^				**0.052**
Unemployed	39 (7.4)	37 (8.2)	2 (2.6)	
Retired	366 (69.3)	304 (67.3)	62 (81.6)	
Inservice	112 (21.3)	100 (22.2)	12 (15.8)	
Student	11 (2.1)	11 (2.4)	0	
BMI^#^	23.44 ± 4.34	23.61 ± 4.32	22.47 ± 4.39	**0.036**
**Comorbidity**				
Hypertension^#^	263 (49.8)	218 (48.2)	45 (59.2)	**0.076**
Diabetes	135 (25.6)	116 (25.7)	19 (25)	0.902
Asthma	28 (5.3)	25 (5.6)	3 (3.9)	0.564
COPD	76 (14.4)	66 (14.7)	10 (13.2)	0.729
Heart failure	39 (7.4)	31 (6.9)	8 (10.5)	0.263
Coronary heart disease	85 (16.1)	74 (16.4)	11 (14.5)	0.666
Liver disease	46 (8.7)	41 (9.1)	5 (6.6)	0.470
Renal disease	95 (18)	83 (18.4)	12 (15.8)	0.112
Cancer	72 (13.6)	58 (12.8)	14 (18.4)	0.189
**Clinical features**^§^				
Cough	406 (76.9)	343 (75.9)	63 (82.9)	0.180
Expectoration	392 (74.2)	345 (76.3)	47 (61.8)	**0.008**
Wheeze	188 (35.6)	141 (31.2)	47 (61.8)	**<0.001**
Fever ≥ 38°C	321 (60.8)	271 (60)	50 (65.8)	0.335
Lymphocyte^#^	1.187 ± 0.762	1.237 ± 0.766	0.884 ± 0.664	**<0.001**
PaO2/FiO2^#^	274.93 ± 114.37	292.74 ± 109.34	209.36 ± 109.22	**<0.001**
Bacteria (+)^*#^	141 (26.7)	45 (59.2)	96 (21.2)	**<0.001**
Multi-viral infection	35 (6.7)	30 (6.7)	5 (6.6)	0.977
Fungi^#^	25 (4.8)	12 (2.7)	13 (17.1)	**<0.001**
Tuberculosis	11 (2.1)	1 (1.3)	10 (2.2)	0.938
Multi-lobular^#^ infiltration	383 (72.5)	72 (94.7)	311 (68.8)	**<0.001**
Early antiviral therapy	166 (31.6)	140 (31.1)	26 (34.2)	0.591
Corticosteroid therapy^#^	198 (37.6)	139 (30.9)	59 (77.6)	**<0.001**
Ventilation				**<0.001**
Non-invasive	75 (14.2)	54 (11.9)	21 (27.6)	
Traumatic	47 (8.9)	18 (4)	29 (38.2)	
ICU	144 (27.3)	95 (21)	49 (64.5)	**<0.001**
Length of hospital stay	20.72 ± 20.35	20.04 ± 19.73	24.79 ± 23.45	**0.037**
Hospitalization cost	55644.36 ± 93022.15	43601.47 ± 81814.74	127267.8 ± 120328.9	**<0.001**

*Continuous parameters presented as mean ± SD, categorical data as n (%). ^∗^Patients with positive sputum or sanguine culture of bacteria were included. §All clinical symptoms were collected on admission. ^
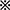
^*P*-value represented the comparison between survival group and death group. ^#^Variables cited in the table above were the candidates which were tested univariably. The bolded values are *p*-values < 0.05, which represent significant differences between survival group and death group. BMI, body mass index; COPD, chronic obstructive pulmonary disease; ICU, intensive care unit.*

In total, 76 (14.4%) died during hospital stay. Significant differences between survivors and non-survivors were shown in Table 1. The median length of stay was 24.79 (SD 23.44) days. There were no statistically significant differences in the early use of antiviral therapy and amount of virus infection between patients survived or dead.

### Immune Responses Among Pneumonia Subgroups

Immune examinations between survival and dead patients are described in Table 2 Lower serum levels of T-lymphocyte subtypes were noted in death group (*p* < 0.01). Moreover, patients from death group were found to possess lower serum levels of T-lymphocyte subtypes (*p* < 0.01) and elevated levels of the cytokines IL-2R (*p* = 0.009), IL-6 (*p* < 0.001) and IL-10 (*p* = 0.033).

**TABLE 2:** Comparison of T-lymphocyte subtypes, humoral immunity and cytokines between bacterial co-infection group and pneumonia severity group.

	**Survival group**	**Overall death**	***p***	**Non-bacteria**	**Co-bacteria**	***p***
T lymphocytes						
CD3 + (%)	68.62 ± 12.63	61.29 ± 17.67	**<0.001**	68.57 ± 13.06	64.60 ± 15.27	**0.020**
CD4 + (%)	40.03 ± 11.19	34.14 ± 14.16	**0.001**	39.67 ± 11.56	37.54 ± 12.70	0.149
CD8 + (%)	26.01 ± 10.74	24.07 ± 15.54	0.271	26.04 ± 11.80	24.85 ± 11.43	0.409
CD3 absolute count	975.35 ± 662.75	530.34 ± 426.76	**<0.001**	1002.81 ± 644.67	666.09 ± 602.81	**<0.001**
CD4 absolute count	466.40 ± 333.71	256.41 ± 191.23	**<0.001**	468.47 ± 318.11	344.52 ± 320.07	**0.008**
CD8 absolute count	328.07 ± 208.02	223.37 ± 231.10	**0.006**	339.19 ± 208.94	245.02 ± 216.68	**0.003**
CD4 + /CD8 + Ratio	1.88 ± 1.09	1.99 ± 1.38	0.536	1.89 ± 1.09	1.91 ± 1.25	0.940
Cytokine						
IL-1	5.69 ± 2.59	7.25 ± 8.34	0.092	6.19 ± 5.41	5.95 ± 3.73	0.763
IL-2R	1290.12 ± 1145.35	1899.69 ± 1323.24	**0.009**	1194.46 ± 1031.01	1732.77 ± 1353.35	**0.008**
IL-6	32.83 ± 74.75	136.22 ± 215.29	**<0.001**	24.74 ± 45.68	95.94 ± 178.97	**0.001**
IL-8	120.51 ± 291.26	207.67 ± 310.19	0.131	115.26 ± 185.37	173.91 ± 392.94	0.239
IL-10	11.12 ± 12.14	16.58 ± 15.72	**0.033**	9.70 ± 10.39	15.81 ± 15.51	**0.005**
TNF-α	15.59 ± 14.53	14.97 ± 10.77	0.828	15.06 ± 14.65	15.85 ± 12.62	0.749

*The bolded values are *p*-values < 0.05, which represent significant differences between subgroups.*

We further analyzed these same immunological indices in patient subgroups with or without bacterial infections. Lower levels of CD3^+^ (*p* < 0.001), CD4^+^ (*p* = 0.008) and CD8^+^ (*p* = 0.003) T-lymphocyte counts were found in the group suffering bacterial infections. As for interleukins, elevated levels of the cytokines IL-2R (*p* = 0.008), IL-6 (*p* = 0.001) and IL-10 (*p* = 0.005) were also found in bacterial group. There were no statistically significant differences in CD4^+^/CD8^+^, IL-1, IL-8 and TNF-α in both comparisons.

### Distribution and Severe Outcome of Bacterial Co-infection

**FIGURE 2:**
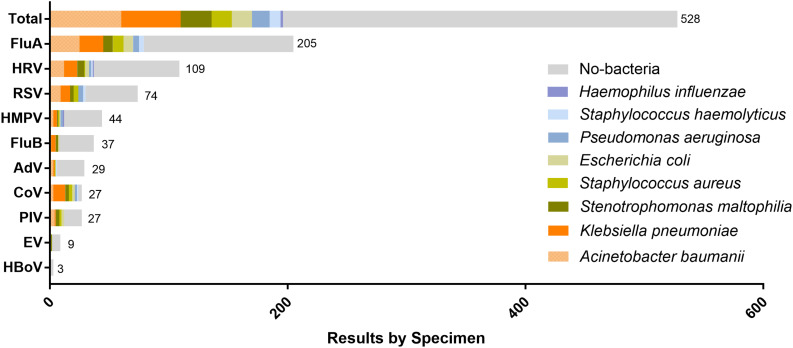
Distribution of respiratory virus and proportion of bacterial infection detected by sputum or blood culture for each virus. The number of patients infected by each virus is presented on the right side of the corresponding horizontal axis.

26.7% hospitalized patients had bacterial coinfection. *Acinetobacter baumannii* was the most commonly isolated pathogen (42.6%, 60/141) followed by *Klebsiella pneumoniae* (35.5%, 50/141), *Stenotrophomonas maltophilia* (18.4%, 26/141), *Staphylococcus aureus* (12.1%, 17/141), *Escherichia coli* (12.1%, 17/141), *Pseudomonas aeruginosa* (10.6%, 15/141), *Haemophilus influenzae* and *Staphylococcus haemolyticus* (Figure 2).

We compared the CURB-65 score, severity and prognoses among patients with or without bacterial co-infection (Table 3). Patients with bacterial infections revealed striking differences in CURB-65 scores, use of either non-invasive or invasive ventilation, ICU admission rate, length of hospitalization and treatment cost as compared with those who simply suffered viral infections.

**TABLE 3:** Comparison of the severity and prognosis between individuals of virus infected pneumonia with or without bacteria co-infection.

	**Non-bacteria, *n* = 387**	**Co-bacteria, *n* = 141**	***p***
CURB-65 score			<0.001
0–1	269 (69.6)	53 (37.6)	
2	87 (22.5)	47 (33.8)	
≥3	31 (8)	41 (29)	
Ventilation			<0.001
Non-invasive	46 (11.9)	31 (22)	
Traumatic	9 (2.3)	38 (27)	
ICU	62 (15.9)	82 (59)	<0.001
Length of stay	15.35 ± 12.38	34.53 ± 28.08	<0.001
Hospitalization cost	29290.51 ± 33255.59	119230.31 ± 119727.77	<0.001

*Continuous parameters presented as mean ± SD, categorical data as n (%).*

### Risk Factors of Mortality

Multiple imputation of missing data was performed for: serum interleukins (28.9% missing); PaO2/FiO2 (25.3% missing); T lymphocyte subsets levels (27.4% missing); body mass index (BMI) and lymphocyte count (all < 1% missing).

According to the methods and analyses above, the following categorical variables were entered in a backward stepwise logistic regression analysis: male; age ≥60 years; smoking history; hypertension; lymphocyte ≤ 0.8^∗^10^9^/L; PO2/FiO2 ≤ 260; IL-6 ≥ 13pg/ml; IL-2R ≥ 1000pg/ml; positive sputum or sanguine culture for bacteria; fungi infection; multilobular infiltration (Table 4).

**TABLE 4:** Univariate analysis associated with mortality of virus-infected pneumonia patients.

**Clinical feature**	**Univariate analysis**	
	
	**Odds Ratio (95% CI)**	***p*-value**
Male	1.553 (0.920–2.624)	0.096
Age ≥ 60	2.266 (1.208–4.250)	0.011
Smoking		0.001
Acute-smoker	2.136 (1.111–4.109)	
Quit-smoker	1.043 (0.829–2.056)	
Non-smoker	1.0	
Hypertension	1.558 (0.951–2.552)	0.072
Lymphocyte ≤ 0.8	5.365 (3.156–9.122)	<0.001
PO2/FiO2 ≤ 260	4.835 (2.557–9.144)	<0.001
IL-6 ≥ 13	3.367 (1.757–6.454)	<0.001
IL-2R ≥ 1000	2.522 (1.315–4.838)	0.005
Bacterial infection	5.383 (3.233–8.963)	<0.001
Fungi infection	7.566 (3.306–17.314)	<0.001
Multilobe infiltrate	6.580 (2.354–18.397)	<0.001

**FIGURE 3:**
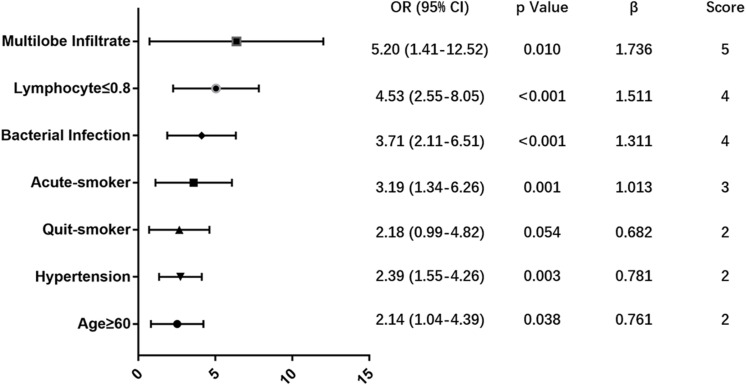
Multivariate analysis associated with mortality of virus-infected pneumonia patients.

**FIGURE 4:**
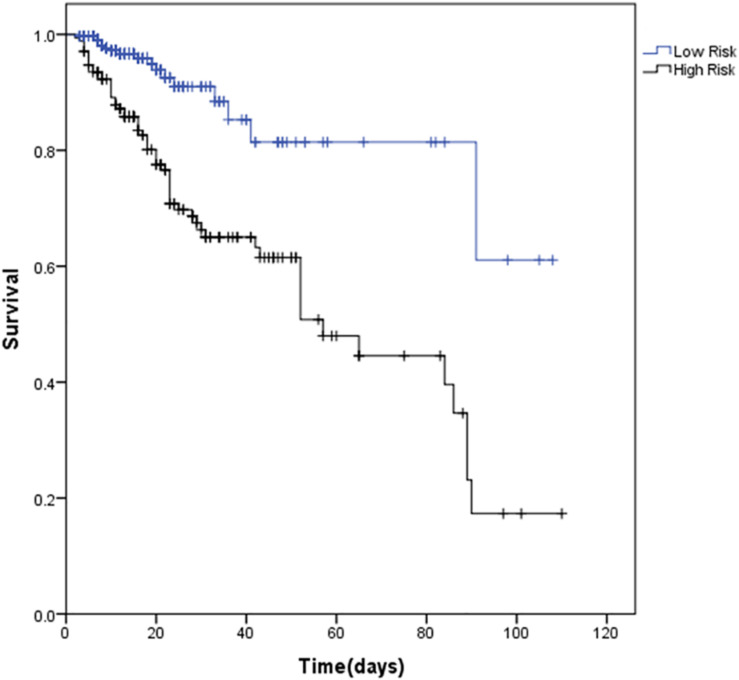
Survival of viral pneumonia patients by different levels of MuLBSTA score (*p* < 0.001). For inhospital mortality: MuLBSTA 0–11 = Low risk; ≥12 = High risk.

In order to develop a simple and useful clinical predicting tool, relative weights were assigned according to the regression coefficient of each categorical variable (β). Figure 3 shows coefficient, odd ratio (OR), 95% CI and calculation of the Multilobular infiltration, hypo-Lymphocytosis, Bacterial coinfection, Smoking history, hyper-Tension and Age (MuLBSTA) Score. AUROC of the training set was 0.821 (95% CI 0.764 to 0.878), and AUROC of the testing set was 0.800 (95% CI 0.683–0.916). For the total 528 patients, AUROC was 0.811 (95% CI 0.76–0.863). Sensitivity, specificity and corresponding risk of death of MuLBSTA are shown in Table 5. Patients were divided in to high-risk and low-risk groups considering the cut-off value of 12. The Kaplan-Meier survival curves for high-risk and low-risk groups are shown in Figure 4.

**TABLE 5:** MuLBSTA score and overall mortality risk.

**Total score**	***n***	**Overall death**	**Estimate of risk (%)**	**Sensitivity**	**Specificity**
0	21	0	0.473	1	0
2	17	0	0.872	1	0.105
3	11	0	1.182	1	0.135
4	25	4	1.601	0.966	0.198
5	23	0	2.165	0.966	0.261
6	11	1	2.921	0.948	0.289
7	41	1	3.930	0.931	0.399
8	20	1	5.271	0.914	0.451
9	60	2	7.033	0.879	0.612
10	14	0	9.329	0.879	0.651
11	41	5	12.274	0.793	0.749
**12**	**11**	**1**	**15.985**	**0.776**	**0.778**
13	50	14	20.555	0.534	0.876
14	17	9	26.027	0.379	0.898
15	19	3	32.363	0.328	0.942
16	5	1	39.419	0.310	0.953
17	20	9	46.945	0.155	0.983
18	6	3	54.613	0.103	0.991
19	4	2	62.068	0.069	0.997
20	5	4	68.994	0	1
21	0	0	NA	NA	NA
22	0	0	NA	NA	NA

*MuLBSTA, multilobular infiltration, hypo-lymphocytosis, bacterial coinfection, smoking history, hyper-tension and age. The bolded values represent the cut-off value of MuLBSTA score and the corresponding estimate of viral pneumonia risk, sensitivity and specificity.*

In comparison, the nomogram of the full regression model in original form is shown in Supplemental Figure 1. Compared with the MuLBSTA score, there was no difference between the AUROC for the original regression model (0.811 vs. 0.847, *p* = 0.19).

**FIGURE 5:**
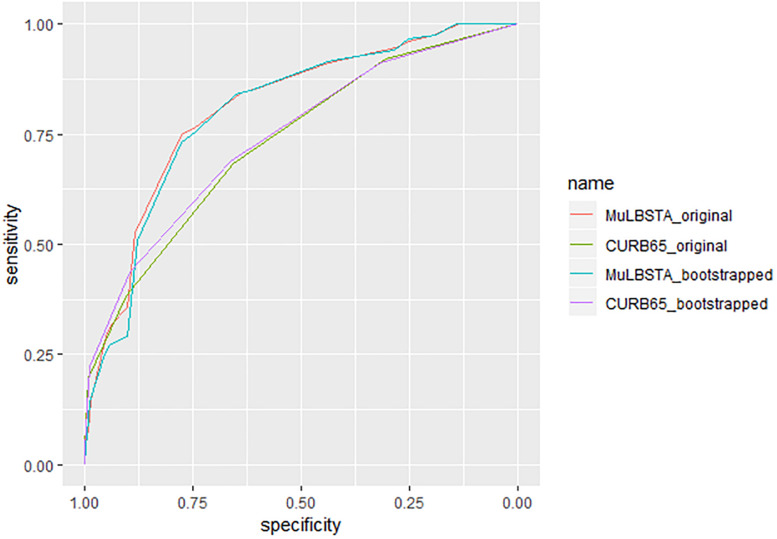
Characteristic curves for prediction of patients with viral pneumonia (*n* = 528). C-index of MuLBSTA score and CURB-65 score are 0.811 and 0.735 separately. The bootstrapped (*n* = 2000) c-index of MuLBSTA and CURB-65 are 0.803 and 0.743.

In our cohort, MuLBSTA was a significantly stronger predictor of overall mortality than CURB-65 (AUROC = 0.811 vs. 0.734, *p* = 0.018, *n* = 528) (Figure 5). The average AUROC of bootstrapped (*n* = 2000) MuLBSTA model and CURB-65 score were 0.806 and 0.728 separately. NRI of MuLBSTA was also improved than CURB-65 (NRI 0.0578, 95% CI 0.0016–0.0865, *p* = 0.04). As CURB-65 was commonly used to predict 30-day mortality, we also assessed the use of MuLBSTA score in 30-day mortality which tended to be a stronger predictor than CURB-65 (AUROC = 0.773 vs. 0.717, *p* < 0.001, *n* = 528).

## Discussion

In patients hospitalized with viral pneumonia, a simple prognostic tool was made for overall mortality which is useful for prediction several days after admission upon obtaining culture results. This score predicts prognoses with greater accuracy than CURB-65.

Pneumonia is a global cause of death with high short-term and long-term mortality. Though short-term mortality rates are high in this acute disease, long-term mortality within 90 days, 1 year and 5 years are also noteworthy in previous studies (Mortensen et al., 2003; Uranga et al., 2018). Nowadays, the survival time for patients with severe lung failure with the progress of radiological image, new drugs and supporting techniques like extracorporeal membrane oxygenation (ECMO) (Pappalardo et al., 2013). A prospective research on viral pneumonia showed a higher 90-day mortality rate than overall mortality as length of hospital stay was between 7 to 14 days (Zhou et al., 2019). During hospitalization, 76 patients in our study died between 4 and 89 days of hospital stay, among them 18 (23.7%) lived longer than 30 days, which makes 90-day mortality worthy of attention.

As immune deficiency is a close relative of mortality, evaluating immune condition could be conductive to monitor patient’s general condition and estimate prognosis. In our study, all T-lymphocyte subtypes were reduced in death group reflecting the deficiency of adaptive immune response. Prior research on viral infection indicated that adaptive T cells provide broader and more lasting cross-reactive cellular immunity with less limitations of strain-specific restriction, especially CD8^+^ T cells (Bender et al., 1992). Besides, the higher level of proinflammatory cytokines had been documented to attribute to severe disease and lung damage (Das et al., 2015). Accordingly, IL-2R and IL-6, which appeared to significantly correlate with illness severity by complementing CD8^+^ T cell function (Nussing et al., 2018), presented with significantly higher serum levels in death group. IL-2R and IL-6 were also found related to mortality in univariate regression. Meanwhile, IL-10 secreted along with adoptive transfer of Th2 CD4^+^ T cell clones, but it was associated with delayed viral clearance and failed to cause protective effect (La Gruta et al., 2007). Although the test of interleukin is not yet widely available, we suggest that patients could be stratified by IL-2R and IL-6 regarding mortality risk.

Bacterial coinfection in the setting of viral pneumonia is known as another major cause of mortality. *Acinetobacter baumannii* is one of the most commonly encountered pathogens both in prior studies and in our investigation (Gao et al., 2013). We further compared patients with or without bacterial infection. Bacterial co-infection not only manifested with worsened outcomes but also prolonged hospital stay and significantly increased the cost of hospital care. Bacterial infection is an independent predictor without other driving forces.

Viral pneumonia further deteriorates when bacterial infection occurs spontaneously. This process is considered to be associated with the dysregulation of T-cell, antigen-specific T cell and plasma cytokine levels (Li and Cao, 2017). Levels of inflammatory cytokines, such as IL-6 and IL-18, were found to be higher in patients suffering bacterial and influenza virus co-infections than in patients infected by a sole pathogen (Li et al., 2012). As such, the remarkably increased IL-6 in patients co-infected with bacteria demonstrated its predictive potential once again.

Despite intense efforts, the development of antiviral therapy to prevent or treat respiratory virus infections is under limitation. Influenza antivirals as oseltamivir or zanamivir were commonly used on the basis of international recommendations (Jefferson et al., 2014). However, oral oseltamivir has a relatively strict time window and several secondary effects like nausea and renal syndromes, and it’s hard to use for unconscious patients (Lee et al., 2017). There is no effective listed antiviral or vaccine approved for the prevention or treatment of non-influenza viruses (Heylen et al., 2017). In our study, oseltamivir was commonly used as antiviral therapy; while acyclovir, ganciclovir or foscarnet were used for cytomegalovirus or herpes simplex virus. Nevertheless, early antiviral treatment did not prevent progression to pneumonia consistent with earlier studies (Elizaga et al., 2001; Chemaly et al., 2012). The confused choice of respiratory virus therapy makes it urgent to predict mortality more accurately.

To date, a variety of studies concerning respiratory viruses were found to demonstrate risk factors by multivariate regression. Consistent with previous report, PO2/FiO2 ≤ 250 in combination with lymphopenia (peripheral blood lymphocyte count <0.8^∗^10^9/^L) were reported to be simple and reliable predictors of influenza (Shi et al., 2017). Multilobular infection was also noted in our study, which was also a remarkable factor in prior report (Jennings et al., 2008). In our study, PO2/FiO2 was also statistically significant mortality predictors according to univariate analysis, while the cut-off was adjusted to 260. Moreover, younger age, chronic comorbid conditions, morbid obesity, high-dose steroid use, hematopoietic stem cell therapy, lower levels of CD4^+^T specific cells and a lack of early antiviral therapy were also regarded as independent risk factors for severe disease, according to prior reports (Viasus et al., 2011; Chemaly et al., 2012; Li and Cao, 2017). However, none of these were significant in our study.

The 2009 IDSA/ATS guidelines had recommended CURB-65 (confusion, urea, respiratory rate, blood pressure, age ≥65 year) as one of CAP severity score (Charles et al., 2009). However, it had a low mortality rate among patients categorized as low risk (Mandell et al., 2007). Several studies argued that increasing age had worse predicting ability due to the fact that influenza A virus had been reported to occur in younger individuals (Riquelme et al., 2011; Bjarnason et al., 2012). Meanwhile, the relative mortality rate of virus infectious diseases in the elderly are reported more than twice those of the young (Pawelec et al., 2002). Early study suggested that a high CD8^+^ T cell count and low NK activity correlated significantly with survival of infectious diseases in the elderly (Ogata et al., 2001), suggesting that aging could lead to increasing immunity deficiency and mortality. In our population of 528 hospitalized viral pneumonia patients, age ≥60 years was statistically associated with mortality while the weight coefficient was relatively small. It is not reasonable to completely deny the importance of age, but appropriate weight adjustment may enhance the predictive capacity of the model.

All parameters identified in the MuLBSTA score are easy to get clinically and all examinations are recommended to be done on admission of hospitalization. ROC and NRI analysis suggests that our new score has better predictive capacity in comparison with CURB-65. Moreover, the MuLBSTA score shows promise for the risk stratification of patients hospitalized with viral pneumonia. the death rates for each grade (Table 5) suggest the following risk categories: MuLBSTA 0-11 (‘low-risk’, mortality = 5.07%); MuLBSTA 12–22 (‘high-risk’, mortality = 33.92%). A higher MuLBSTA score might be used as a good predictor of prognosis.

Some limitations of this study should also be acknowledged. The retrospective single-center design leads to missing data and unavoidable biases in identifying and recruiting participants. The sample size was relatively small in order to build up a predicting score. Despite these limitations, the study was designed to reflect the ‘real life’ clinical situation. Clinical information was meticulously gathered using standard protocols by admitted medical team. This score might assist clinicians in making appropriate decisions and optimizing the use of hospital resources.

## Conclusion

We found that the MuLBSTA score, based on six parameters routinely available in hospital, has a strong predictive ability for 90-day mortality. It can accurately stratify hospitalized patients with viral pneumonia into relevant risk categories and could provide guidance to make further clinical decisions.

## Data Availability Statement

The datasets analyzed for this study can be found in the Figshare. Link: https://figshare.com/articles/dataset_for_the_MuLBSTA_score_xlsx/10333475.

## Ethics Statement

This study was approved by the Coordinating Ethics Committee of Ruijin Hospital Affiliated to Shanghai Jiao Tong University School of Medicine (No. 2017-205). Written informed consents were obtained from all patients involved before enrolment. In our study, patients from 18 to 96 years old were included. The consent obtained from the participants was both informed and written.

## Author Contributions

MZ and XZ conceived or designed the work. LG, DW, and YW collected the data. LG, DW, and QL analyzed and interpreted the data. LG, DW, QL, and JQ drafted the manuscript. All authors critically revised the manuscript and approved the final version of the manuscript to be published.

## Conflict of Interest

The authors declare that the research was conducted in the absence of any commercial or financial relationships that could be construed as a potential conflict of interest.

## Abbreviations

AdV: adenovirus
ARDS: acute respiratory distress syndrome
AUROC: area under ROC curve
CAP: community acquired pneumonia
CoV: coronavirus
CURB-65: confusion, urea, blood pressure, respiratory rate, age ≥ 65 year
ECMO: extracorporeal membrane oxygenation
EV: enterovirus
FluA: influenza A
FluB: influenza B
HMPV: human metapneumovirus
HRV: human rhinovirus
ICU: intensive care unit
IL: interleukin
MuLBSTA Score: multilobular infiltration, hypo-lymphocytosis, bacterial coinfection, smoking history, hyper-tension and age
NRI: net reclassification improvement
PIV: parainfluenza
ROC: receiver operator characteristic
RSVA: respiratory syncytial virus A
RSVB: respiratory syncytial virus B
RT-PCR: reverse-transcription polymerase chain reaction
TNF-α: tumor necrosis factor- α.

## References

[B1] Barlow G. Nathwani D. Davey P. (2007). The CURB65 pneumonia severity score outperforms generic sepsis and early warning scores in predicting mortality in community-acquired pneumonia. *Thorax* 62 253–259. 10.1136/thx.2006.067371 16928720 PMC2117168

[B2] Bender B. S. Croghan T. Zhang L. Small P. A. Jr. (1992). Transgenic mice lacking class I major histocompatibility complex-restricted T cells have delayed viral clearance and increased mortality after influenza virus challenge. *J. Exp. Med.* 175 1143–1145. 10.1084/jem.175.4.1143 1552285 PMC2119177

[B3] Bjarnason A. Thorleifsdottir G. Löve A. Gudnason J. F. Asgeirsson H. Hallgrimsson K. L. (2012). Severity of influenza A 2009 (H1N1) pneumonia is underestimated by routine prediction rules. Results from a prospective, population-based study. *PloS One* 7:e46816. 10.1371/journal.pone.0046816 23071646 PMC3469650

[B4] Charles P. G. Davis J. S. Grayson M. L. (2009). Rocket science and the Infectious Diseases Society of America/American Thoracic Society (IDSA/ATS) guidelines for severe community-acquired pneumonia. *Clin. Infect. Dis.* 48:1796. 10.1086/599227 19473089

[B5] Chemaly R. F. Hanmod S. S. Rathod D. B. Ghantoji S. S. Jiang Y. Doshi A. (2012). The characteristics and outcomes of parainfluenza virus infections in 200 patients with leukemia or recipients of hematopoietic stem cell transplantation. *Blood* 119 2738–2745. 10.1182/blood-2011-08-371112 22246027

[B6] Das D. Le Floch H. Houhou N. Epelboin L. Hausfater P. Khalil A. (2015). Viruses detected by systematic multiplex polymerase chain reaction in adults with suspected community-acquired pneumonia attending emergency departments in France. *Clin. Microbiol. Infect.* 21 e1–e8. 10.1016/j.cmi.2015.02.014 25704448 PMC7128919

[B7] Elizaga J. Olavarria E. Apperley J. Goldman J. Ward K. (2001). Parainfluenza virus 3 infection after stem cell transplant: relevance to outcome of rapid diagnosis and ribavirin treatment. *Clin. Infect. Dis.* 32 413–418. 10.1086/318498 11170949

[B8] Gao H. N. Lu H. Z. Cao B. Du B. Shang H. Gan J. H. (2013). Clinical findings in 111 cases of influenza a (H7N9) virus infection. *N. Engl. J. Med.* 368 2277–2285. 10.1056/NEJMoa1305584 23697469

[B9] Hanada S. Pirzadeh M. Carver K. Y. Deng J. C. (2018). Respiratory Viral Infection-Induced Microbiome Alterations and Secondary Bacterial Pneumonia. *Front. Immunol.* 9:2640. 10.3389/fimmu.2018.02640 30505304 PMC6250824

[B10] Heylen E. Neyts J. Jochmans D. (2017). Drug candidates and model systems in respiratory syncytial virus antiviral drug discovery. *Biochem. Pharmacol.* 127 1–12. 10.1016/j.bcp.2016.09.014 27659812

[B11] Jefferson T. Jones M. Doshi P. Spencer E. A. Onakpoya I. Heneghan C. J. (2014). Oseltamivir for influenza in adults and children: systematic review of clinical study reports and summary of regulatory comments. *BMJ Apr.* 9:348. 10.1136/bmj.g2545 24811411 PMC3981975

[B12] Jennings L. C. Anderson T. P. Beynon K. A. Chua A. Laing R. T. Werno A. M. (2008). Incidence and characteristics of viral community-acquired pneumonia in adults. *Thorax* 63 42–48. 10.1136/thx.2006.075077 17573440

[B13] La Gruta N. L. Kedzierska K. Stambas J. Doherty P. C. (2007). A question of self-preservation: immunopathology in influenza virus infection. *Immunol. Cell Biol.* 85 85–92. 10.1038/sj.icb.7100026 17213831

[B14] Lee J. Park J. H. Jwa H. Kim Y. H. (2017). Comparison of Efficacy of Intravenous Peramivir and Oral Oseltamivir for the Treatment of Influenza: Systematic Review and Meta-Analysis. *Yonsei Med. J.* 58 778–785. 10.3349/ymj.2017.58.4.778 28540991 PMC5447109

[B15] Leening M. J. Vedder M. M. Witteman J. C. Pencina M. J. Steyerberg E. W. (2014). Net reclassification improvement: computation, interpretation, and controversies: a literature review and clinician’s guide. *Ann. Intern. Med.* 160 122–131. 10.7326/M13-1522 24592497

[B16] Li H. Cao B. (2017). Pandemic and Avian Influenza A Viruses in Humans: Epidemiology, Virology, Clinical Characteristics, and Treatment Strategy. *Clin. Chest Med.* 38 59–70. 10.1016/j.ccm.2016.11.005 28159162

[B17] Li W. Moltedo B. Moran T. M. (2012). Type I interferon induction during influenza virus infection increases susceptibility to secondary Streptococcus pneumoniae infection by negative regulation of gammadelta T cells. *J. Virol.* 86 12304–12312. 10.1128/JVI.01269-12 22951826 PMC3486468

[B18] Mandell L. A. Wunderink R. G. Anzueto A. Bartlett J. G. Campbell G. D. Dean N. C. (2007). Infectious Diseases Society of America/American Thoracic Society consensus Guidelines on the Management of Community–Acquired Pneumonia in Adults. *Clin. Infect. Dis.* 44(Suppl. 2), S27–S72. 10.1086/511159 17278083 PMC7107997

[B19] Mortensen E. M. Kapoor W. N. Chang C. C. Fine M. J. (2003). Assessment of mortality after long-term follow-up of patients with community-acquired pneumonia. *Clin. Infect. Dis.* 37 1617–1624. 10.1086/379712 14689342

[B20] Nussing S. Sant S. Koutsakos M. Subbarao K. Nguyen T. H. O. Kedzierska K. (2018). Innate and adaptive T cells in influenza disease. *Front. Med.* 12 34–47. 10.1007/s11684-017-0606-8 29352371

[B21] Ogata K. An E. Shioi Y. Nakamura K. Luo S. Yokose N. (2001). Association between natural killer cell activity and infection in immunologically normal elderly people. *Clin. Exp. Immunol.* 124 392–397. 10.1046/j.1365-2249.2001.01571.x 11472399 PMC1906081

[B22] Pappalardo F. Pieri M. Greco T. Patroniti N. Pesenti A. Arcadipane A. (2013). Predicting mortality risk in patients undergoing venovenous ECMO for ARDS due to influenza A (H1N1) pneumonia: the ECMOnet score. *Intensive Care Med.* 39 275–281. 10.1007/s00134-012-2747-1 23160769 PMC7095375

[B23] Pawelec G. Barnett Y. Forsey R. Frasca D. Globerson A. McLeod J. (2002). T cells and aging, January 2002 update. *Front. Biosci.* 7 d1056–d1183. 10.2741/a831 11991846

[B24] Riquelme R. Jimenez P. Videla A. J. Lopez H. Chalmers J. Singanayagam A. (2011). Predicting mortality in hospitalized patients with 2009 H1N1 influenza pneumonia. *Int. J. Tuberc. Lung. Dis.* 15 542–546. 10.5588/ijtld.10.0539 21396216

[B25] Shi S. J. Li H. Liu M. Liu Y. M. Zhou F. Liu B. (2017). Mortality prediction to hospitalized patients with influenza pneumonia: PO2/FiO2 combined lymphocyte count is the answer. *Clin. Respir. J.* 11 352–360. 10.1111/crj.12346 26148709 PMC7162301

[B26] Shorr A. F. Zilberberg M. D. Micek S. T. Kollef M. H. (2017). Viruses are prevalent in non-ventilated hospital-acquired pneumonia. *Respir. Med.* 122 76–80. 10.1016/j.rmed.2016.11.023 27993295 PMC7135153

[B27] Sterne J. A. White I. R. Carlin J. B. Spratt M. Royston P. Kenward M. G. (2009). Multiple imputation for missing data in epidemiological and clinical research: potential and pitfalls. *BMJ* 29:b2393. 10.1136/bmj.b2393 19564179 PMC2714692

[B28] Uranga A. Quintana J. M. Aguirre U. Artaraz A. Diez R. Pascual S. (2018). Predicting 1-year mortality after hospitalization for community-acquired pneumonia. *PloS One* 13:e0192750. 10.1371/journal.pone.0192750 29444151 PMC5812619

[B29] Viasus D. Del Rio-Pertuz G. Simonetti A. F. Garcia-Vidal C. Acosta-Reyes J. Garavito A. (2016). Biomarkers for predicting short-term mortality in community-acquired pneumonia: a systematic review and meta-analysis. *J. Infect.* 72 273–282. 10.1016/j.jinf.2016.01.002 26777314

[B30] Viasus D. Pano-Pardo J. R. Pachon J. Campins A. López-Medrano F. Villoslada A. (2011). Factors associated with severe disease in hospitalized adults with pandemic (H1N1) 2009 in Spain. *Clin. Microbiol. Infect.* 17 738–746. 10.1111/j.1469-0691.2010.03362.x 20825436

[B31] Zhou F. Wang Y. Liu Y. Liu X. Gu L. Zhang X. (2019). Disease severity and clinical outcomes of community-acquired pneumonia caused by non-influenza respiratory viruses in adults: a multicentre prospective registry study from the CAP-China Network. *Eur. Respir. J.* 54:1802406. 10.1183/13993003.02406-2018 31164430

