# Erratum: Clinical Features Predicting Mortality Risk in Patients With Viral Pneumonia: The MuLBSTA Score

**DOI:** 10.3389/fmicb.2020.01304

**Published:** 2020-06-09

**Authors:** Frontiers Production Office

**Affiliations:** Frontiers Media SA, Lausanne, Switzerland

**Keywords:** virus pneumonia, predicting mortality, bacterial coinfection, predictive score model, clinical feature

Due to a production error, the author “Jieming Qu” (jmqu0906@163.com) was not included in the Correspondence section. The publisher apologizes for this mistake.

The original article has been updated.

