# Supplementary material for: Clinical Features Predicting Mortality Risk in Patients With Viral Pneumonia: The MuLBSTA Score
Source: Front Microbiol. 2019 Dec 3;10:2752. doi: 10.3389/fmicb.2019.02752 (PMC6901688; doi:10.3389/fmicb.2019.02752)
Supplement: Supplementary file 1 [file Data_Sheet_1.docx]

Supplemental Materials


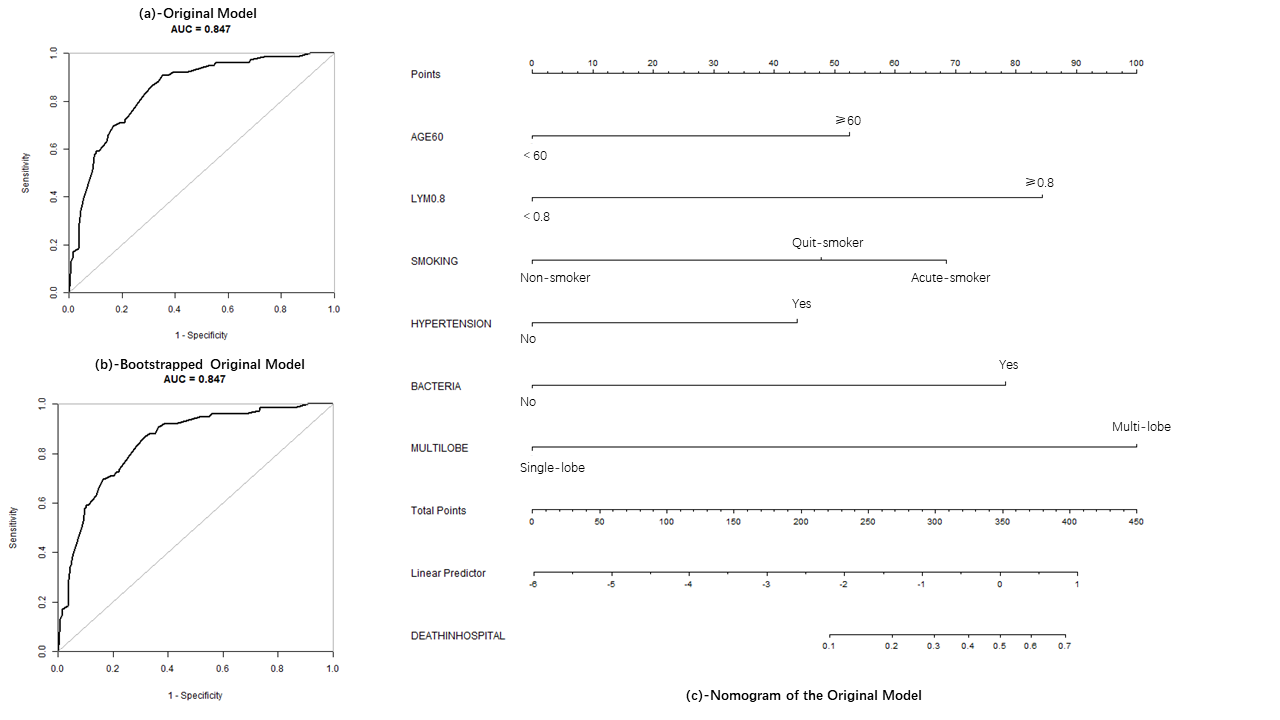


Supplemental figure 1. Original or bootstrapped multivariate logistic regression model associated with mortality of viral pneumonia patients. (a): AUROC of the full regression model in original form; (b): AUROC of the bootstrapped regression model (n=2000) with all variables in original form; (c): nomogram of the full regression model in original form.
